# Permissiveness of bovine epithelial cells from lung, intestine, placenta and udder for infection with *Coxiella burnetii*

**DOI:** 10.1186/s13567-017-0430-9

**Published:** 2017-04-12

**Authors:** Katharina Sobotta, Katharina Bonkowski, Elisabeth Liebler-Tenorio, Pierre Germon, Pascal Rainard, Nina Hambruch, Christiane Pfarrer, Ilse D. Jacobsen, Christian Menge

**Affiliations:** 1 Institute of Molecular Pathogenesis, Friedrich-Loeffler-Institut (FLI), Naumburger Strasse 96a, 07743 Jena, Germany; 2 ISP, INRA, Université Tours, UMR 1282, 37380 Nouzilly, France; 3 Department of Anatomy, University of Veterinary Medicine Hannover, Bischofsholer Damm 15, 30173 Hannover, Germany; 4 Research Group Microbial Immunology, Leibniz Institute for Natural Product Research and Infection Biology/Hans Knoell Institute, Beutenbergstrasse 11a, 07745 Jena, Germany

## Abstract

Ruminants are the main source of human infections with the obligate intracellular bacterium *Coxiella* (*C.*) *burnetii*. Infected animals shed high numbers of *C. burnetii* by milk, feces, and birth products. In goats, shedding by the latter route coincides with *C. burnetii* replication in epithelial (trophoblast) cells of the placenta, which led us to hypothesize that epithelial cells are generally implicated in replication and shedding of *C. burnetii*. We therefore aimed at analyzing the interactions of *C. burnetii* with epithelial cells of the bovine host (1) at the entry site (lung epithelium) which govern host immune responses and (2) in epithelial cells of gut, udder and placenta decisive for the quantity of pathogen excretion. Epithelial cell lines [PS (udder), FKD-R 971 (small intestine), BCEC (maternal placenta), F3 (fetal placenta), BEL-26 (lung)] were inoculated with *C. burnetii* strains Nine Mile I (NMI) and NMII at different cultivation conditions. The cell lines exhibited different permissiveness for *C. burnetii.* While maintaining cell viability, udder cells allowed the highest replication rates with formation of large cell-filling Coxiella containing vacuoles. Intestinal cells showed an enhanced susceptibility to invasion but supported *C. burnetii* replication only at intermediate levels. Lung and placental cells also internalized the bacteria but in strikingly smaller numbers. In any of the epithelial cells, both Coxiella strains failed to trigger a substantial IL-1β, IL-6 and TNF-α response. Epithelial cells, with mammary epithelial cells in particular, may therefore serve as a niche for *C. burnetii* replication in vivo without alerting the host’s immune response.

## Introduction

*Coxiella* (*C.*) *burnetii* is a Gram-negative, obligate intracellular pathogen and causative agent of Q fever, a widely distributed zooanthroponosis [1]. The disease appears as an acute, flu-like and self-limiting illness, or manifests as a chronically progressing infection (e.g., endocarditis, premature delivery in pregnant women). *C. burnetii* has a broad host spectrum, which includes birds, reptiles, arthropods and domestic and wild mammals. Sources of human infections often are infected sheep, goats or cattle [2]. In livestock, *C. burnetii* infection is inapparent in most cases [1]. If disease manifests, referred to as Coxiellosis, reproductive disorders such as abortions, stillbirth in goats and sheep or delivery of weak newborns in cattle were observed [3]. The main route of *C. burnetii* transmission is via inhalation of infected aerosols or dust, especially when contaminated with *C. burnetii* birth products (placental membranes and fluids) of goat and sheep, but also by feces and milk [4, 5]. An unprecedented large Q-fever outbreak occurred from 2007 to 2011 in the Netherlands, where more than 4000 human cases were notified and approximately 52 000 ruminants were culled as part of the countermeasures taken to control the epidemic [6].

Main shedding of *C. burnetii* with about 10^9^ bacteria per gram placenta is observed during parturition in sheep and goat [1]. Coxiella organisms are primarily detected in trophoblast cells in the placentomes [2, 7, 8]. Shedding of bacteria by milk of asymptomatic cattle was observed to persist for several months [9]. Dairy cows seem to be more chronically infected with *C. burnetii* than small ruminants. Guatteo et al. [10] could also show that Coxiella shedding was scarce and sporadic in feces of cattle, whereas permanent and sporadic shedding was observed by milk. PCR analysis of bovine bulk tank milk samples detected more than 10^2^ *C. burnetii* DNA equivalents per milliliter [11]. Muskens et al. [12] believe that the localization of the pathogen in the bovine udder is the critical factor for a secretion of bacteria into the milk but it is currently unknown which cell types facilitate persistence and replication of *C. burnetii* in the mammary gland.

The chain of events implicated in *C. burnetii* transmission between animals of the reservoir species have poorly been studied at the cellular level. Mononuclear phagocytes, e.g., macrophages and monocytes, are considered the major host cells during natural infection [1]. We recently showed that Coxiella organisms invade primary bovine monocyte-derived and alveolar macrophages in vitro and slowly replicate in these cells without significantly activating them [13]. Even though alveolar macrophages probably represent the first target cell for *C. burnetii* when entering the body, it is highly likely that alveolar epithelial cells also become exposed to and infected by these bacteria in the early stages of the infection as described in rodent models [14, 15]. At the site of entry, the lung epithelium therefore may determine the character of the subsequent immune response in concert with alveolar macrophages. It is also perceivable that epithelial cells at the exit sites are determinative for persistence in and bacterial transmission of *C. burnetii* from the reservoir host [1, 4].

In general, *C. burnetii* is able to grow in a number of cell types, like Vero cells or fibroblast cells [1]. All these cell types poorly mirror the natural cell environment to investigate infection processes in domestic animals. Therefore the present study aimed at developing an in vitro cell system deploying bovine epithelial cell lines from lung, placenta, gut and udder tissues. We studied the permissiveness and host cell response of different epithelial cells with two Coxiella strains: a virulent strain (Nine Mile I), expressing full-length lipopolysaccharide (“smooth LPS”), and an avirulent strain (NM phase II). NMII lacks the full O-polysaccharide I chain and sugars located in the LPS I outer core (called “rough LPS”) [16].

## Materials and methods

### Epithelial cell culture

Source, origin and characteristics of bovine epithelial cells used in this study are provided in Table 1. The epithelial origin of all cell lines deployed was confirmed by detection of cytokeratin and zonula occludens protein (ZO-2) via fluorescence microscopy and western blot analysis (data not shown). Cell culture media were obtained from Life Technologies (Darmstadt, Germany). The basis culture medium of the bovine udder epithelial cells (PS) was supplemented with Insulin-like growth factor 1 (10 ng/mL; Peprotech, Hamburg, Germany), Fibroblast growth factor (5 ng/mL Peprotech), Epidermal growth factor (5 ng/mL; Sigma, Taufkirchen, Germany), Hydrocortisone (1 µg/mL; Sigma), 20 mM HEPES buffer (Fisher Scientific, Schwerte, Germany) and 2 mM l-Glutamine (Life Technologies) [17]. Maintenance media were supplemented with 10% fetal bovine serum [FBS, Fisher Scientific; heat-inactivated (56 °C for 30 min)]. Test media used in experiments for invasion analysis, cell vitality test and cytokine analysis contained 1% FBS. Epithelial cells were grown and maintained in an atmosphere of 5% CO_2_ at 37 °C. Detachment of the cells was done with Trypsin/EDTA solution [NaCl 0.8% (w/v), KCl 0.08% (v/v), Dextrose 0.1% (w/v), Na_2_HCO_3_ 0.058% (w/v), Trypsin 0.05% (w/v), EDTA 0.02% (w/v)] for 5 min at 5% CO_2_ at 37 °C. Cells were seeded into 96-, 24- or 6-well plates with 1 × 10^4^, 4–10 × 10^4^ and 2–3 × 10^5^ cells, respectively, and cultured for 2–3 days to reach a confluent cell monolayer.

**Table 1:** **Cell lines used in this study**

Code	Clone	Origin		Culture medium	Source	Reference
		Organ	Donor			
L	BEL-26	Lung	Fetal	Dulbecco’s Modified Eagle Medium (DMEM, 1.0 g/mL glucose)	CCLV^a^	(–)
G	FKD-R 971	Jejunum	Fetal	Iscove’s Modified Dulbecco’s Medium (IMDM)/Ham’s F12 nutrient mix [1:1]	CCLV^a^	(–)
Pm	BCEC	Maternal placenta	Mature/pregnant	DMEM (4.5 g/mL glucose)/Ham’s F12 nutrient mix [1:1]	TiHo^b^	[35]
Pf	F3	Fetal placenta	Mature/pregnant	DMEM (4.5 g/mL glucose)/Ham’s F12 nutrient mix [1:1]	TiHo^b^	[36]
U	PS	Udder	Mature	Advanced DMEM/Ham’s F12 nutrient mix [1:1] + additional components^d^	INRA^c^	[17]

^a^Collection of Cell Lines in Veterinary Medicine (CCLV) at Friedrich-Loeffler-Institut (Isle of Riems), Germany; cell line FKD-R 971 was generated by Roland Riebe (RIE 971).

^b^Christiane Pfarrer, Department of Anatomy, University of Veterinary Medicine, Hannover, Germany.

^c^Pascal Rainard, ISP, INRA, Université Tours, Nouzilly, France.

^d^See “Materials and methods”.

### *Coxiella* (*C.*) *burnetii* infection and sampling

Cells were infected with *C. burnetii* strain “Nine Mile phase I RSA 493” (NMI) and “Nine Mile phase II clone 4” (NMII) (phase I-LPS and phase II-LPS expressing variants, respectively) in multi-well cell culture plates at 37 °C and 5% CO_2_. Both *C. burnetii* strains were supplied, propagated and purified as previously described [13].

**Figure 1:**
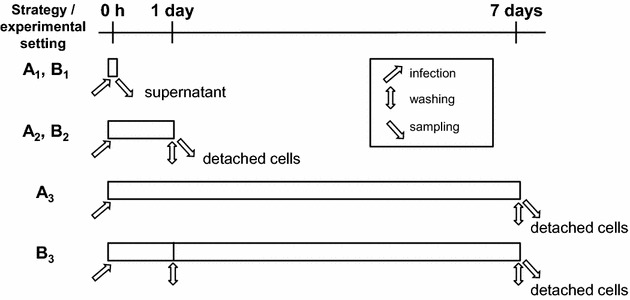
**Study design.** Invasion and replication of *C. burnetii* in bovine epithelial cell lines were quantified applying two different infection strategies differing in the time the inocula were left with the cells. Strategy B included a washing step after 1 day; in strategy A, inocula were not removed for the duration of the experiment. “Invasion B” refers to the quantitation of cell-associated *C. burnetii* genome equivalents (GE) 24 h after inoculation (values obtained in experimental setting B_2_), “invasion A” to the quantitation of cell-associated GE after 7 days (values obtained in experimental setting A_3_), each normalized to the GE numbers detected in the supernatant regained immediately after inoculation (time point “0 h”; values obtained in experimental settings A_1_ and B_1_). Thus the invasion was calculated as follows: (A) x = (A_3_ × 100%)/A_1_ and (B) x = (B_2_ × 100%)/B_1_. Replication efficiency was calculated as the fold-increase in the number of cell-associated GE from day 1 (values obtained in experimental setting A_2_ and B_2_) to values at day 7 (obtained in experimental setting A_3_ and B_3_). Thus the replication efficiency was calculated as follows: (A) = A_3_/A_2_ and (B) = B_3_/B_2_. Arrow depict sampling event, i.e. taking off the supernatant, washing the cell monolayer and detachment of the cells.

Once cells had formed a monolayer, cell numbers from a reference well were determined by detaching with Trypsin/EDTA solution and subsequent microscopic cell counting with a Neubauer chamber. Cells were infected with a multiplicity of infection (MOI) of 100 or mock infected with NaCl solution. *C. burnetii* inocula were left in the cultures for 7 days (infection strategy A; Figure 1). Alternatively, non-internalized bacteria were removed 24 h after inoculation by washing two to three times with pre-warmed (37 °C) 1× PBS and fresh culture test medium (1% FBS) was added (infection strategy B). During each set of experiments, supernatants (extracellular fraction) and cells (cell-associated fraction) were sampled directly after inoculation at time point “0 h” (“regained inoculum”; experimental settings A_1_, B_1_), after 24 h (“1 day”; experimental settings A_2_, B_2_) or after 7 days (“7 days”; (experimental settings A_3_ and B_3_) from separate wells. To this end, wells were washed two to three times with warm 1× PBS and cells were detached by incubation with Trypsin/EDTA solution. To inactivate *C. burnetii*, samples were treated with three freeze (−80 °C)/thaw cycles and subsequently incubated for 30 min at 95 °C. For detection of host cell immune response, cells were inoculated following infection strategy B_2_ or B_3_, or stimulated with LPS of *E. coli* O111:B4 (5 µg/mL) as a control. Cells were harvested for RNA isolation 1 and 7 days post-infection (pi). Total RNA was extracted with RNeasy Mini Kit (Qiagen, Hilden, Germany) according to the instructions of the manufacturer. To avoid DNA contamination RNA was purified with the RNase-free DNase set (Qiagen). For immunofluorescence microscopy studies, infected cells were inoculated following strategy B_3_.

### Immunofluorescence microscopy

Cells were cultured in 24-well plates (Corning^®^ Costar^®^, Sigma) until at least 80% confluence was reached and infected with NMI and NMII with a MOI of 100 as described above. Cell vitality was monitored by MTT and LDH assay (according to manufacturer’s instructions). After 7 days of infection (see above), cells were fixed with 4% formaldehyde for 24 h and stored at 4 °C. Cells were washed with 1× PBS and permeabilized with 100% ice cold methanol for 1 min followed by incubation with 50 nM NH_4_Cl for 15 min and PBS/FBS solution (1% FBS) for 30–45 min in the dark at room temperature to block unspecific binding sites. Cells were stained for 30 min at room temperature using an Anti-Coxiella antibody [1:5000 in PBS/FCS] (kindly provided by Anja Lührmann, Friedrich-Alexander-Universität Erlangen-Nürnberg, Germany) followed by incubation (30 min–1 h) with a fluorochrome-conjugated secondary antibody [1:500 in PBS/FCS] [Anti-rabbit IgG (H + L), F(ab’) 2 Fragment (Alexa Fluor^®^ 594 Conjugate), New England Biolabs, Frankfurt, Germany], counterstained with 4′,6-diamidino-2-phenylindole (DAPI) for 5 min at room temperature followed by washing with 1× PBS. To characterize the replication compartment of *C. burnetii*, infected cells were labeled with the acidic marker LysoTracker Red DND-99 (Invitrogen, Darmstadt, Germany). After infection, cells were incubated with LysoTracker (1:5000 in test media) for 2 h at 37 °C following fixation as described above. The cells were mounted in DABCO (1,4-diazabicyclo[2.2.2]-octane, 2% in Glycerol). Samples were viewed under a fluorescence microscope [Olympus CK40; camera: Leica DFC420C, software: Leica Application Suite (LAS) Version 3.7.0 (Build: 681)]. Cultures incubated in PBS without primary antibodies served as negative controls.

### Transmission electron microscopy (TEM)

Cells were cultured in 6-well plates (Corning^®^ Costar^®^, Sigma) and infected with infection strategy B_3_ as described above. The culture supernatant was removed and cells were fixed with 2.5% glutaraldehyde in cacodylate buffer (0.1 M, pH 7.2) for 2 h at 4 °C, detached with a cell scraper from the culture plate, collected in a reaction tube and centrifuged to obtain a cell pellet [18]. The cell pellet was embedded in 2% agarose and sectioned to 1 mm^3^ cubes. Cubes were post fixed in 2% osmium tetroxide and embedded in araldite Cy212. Ultrathin sections (85 nm) were stained with uranyl acetate and lead citrate. They were examined at an accelerating voltage of 80 kV by transmission electron microscopy (Tecnai12, FEI, Eindhoven, Netherlands).

### Quantitative real-time PCR for determination of intracellular *C. burnetii* genome equivalents (GE)

To estimate the number of cell associated bacteria, DNA from the cell containing fraction of the cultures of infection strategy A and B was purified with the Invisorb^®^ DNA Cleanup-Kit (Stratec, Birkenfeld, Germany) according to manufacturer’s instructions. The number of genome equivalents (GE) was monitored by quantitation of the isocitrate dehydrogenase (*icd*) gene by quantitative real-time PCR (qPCR) [19]. The *icd* gene is highly conserved within the species *C. burnetii* and occurs as single copy in the Coxiella genome. Ct values of technical duplicates varied by less than 0.51 and were used to calculate GE considering values obtained with an entrained *icd* harbouring plasmid standard. Invasion rate and replication efficiency were calculated from four biological replicates (independent cell cultures, tested in two technical replicates each) as described in the legend to Figure 1.

### Flow cytometry analysis

Cells (4 × 10^5^ cells/well, 24 well plates) were detached by Trypsin/EDTA solution and transferred to microtiter plates (V-shape; Greiner Bio-One GmbH, Frickenhausen, Germany) and pelletized by centrifugation (400 × *g*, 4 min and 4 °C). For detection of CR3 [CD11b, MM12A, diluted 1:250 (VMRD, Pullman, WA, USA)] and α_V_β_3_ [CD61, diluted 1:100 (AbD Serotec, Düsseldorf, Germany)], cells were incubated with 50 µL diluted primary antibody for 20 min. After washing (washing buffer: 1× PBS, 0.5% FCS), cells were incubated with secondary antibody anti-mouse IgG1-APC (Southern Biotech, Birmingham, USA) diluted 1:1000 in 1× PBS for 20 min. Finally cells were washed again and analyzed with BD FACSCanto™II (Becton–Dickinson, Heidelberg, Germany). Data was analyzed with BD FACSDIVA™ software (version 6).

### Reverse transcription and cytokine-specific real time PCR

Equal amounts of RNA from each sample were reversely transcribed into cDNA as described previously [13]. Levels of relative gene expression of different cytokines in comparison to GAPDH as housekeeping gene were determined by quantitative real-time SYBR Green-based (Applied Biosystems, Waltham, USA) PCR (qPCR) using ABI Prism^®^7500 (Applied Biosystems). All primers (Table 2) were run at an annealing temperature of 60 °C. The reaction profile applied was: denaturation (10 min, 95 °C), annealing (1 min, 60 °C; 39 cycles) and melting step (15, 60 °C). Ct values for GAPDH-specific mRNA were not subject to variation along the incubation period and values from infected cells did not differ from non-infected control cells (data not shown). Relative gene expression levels were calculated by using relative expression software REST [20].

**Table 2:** **Sequences of primers used in this study**

Primer	Sequence 5′–3′
GAPDH	F: GCG ATA CTC ACT CTT CTA CCT TCG A R: TCG TAC CAG GAA ATG AGC TTG AC
IL-1β	F: ACC TGA ACC CAT CAA CGA AAT G R: TAG GGT CAT CAG CCT CAA ATA ACA
IL-6	F: CTG AAG CAA AAG ATC GCA GAT CTA R: CTC GTT TGA AGA CTG CAT CTT CTC
TNF-α	F: TCT TCT CAA GCC TCA AGT AAC AAG T R: CCA TGA GGG CAT TGG CAT AC

### Statistical analysis

Unless otherwise indicated, Mann–Whitney U test was used to compare two different samples. A *p* value of ≤0.05 (“a” or “*”) show a statistically significant difference at the 95% confidence level, a *p* value of ≤0.01 (“b” or “**”) at the 99% confidence level.

## Results

### Bovine udder epithelial cells exhibited highest permissiveness for *C. burnetii* propagation

**Figure 2:**
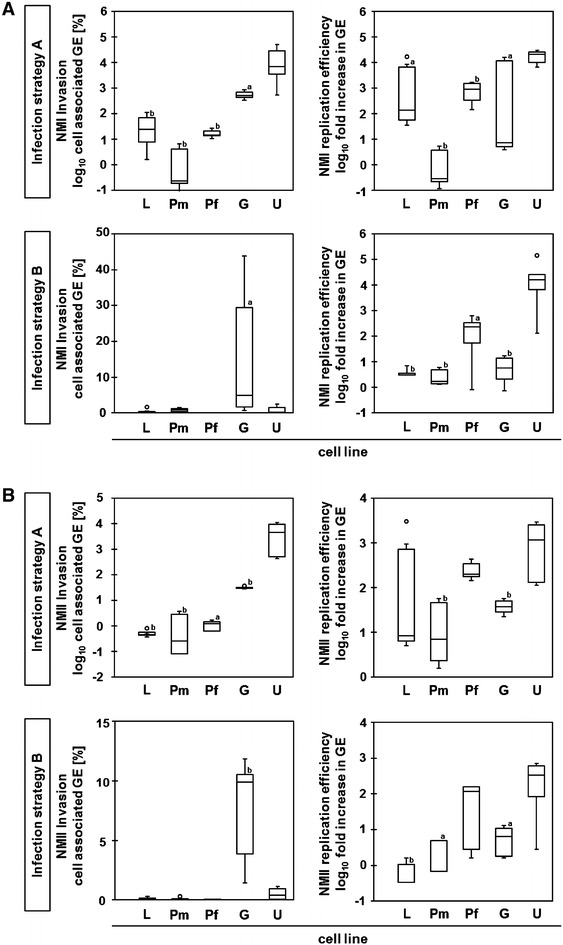
**Invasion and replication efficiency of** ***C.*** ***burnetii*** **in different bovine epithelial cell lines.** Efficiencies were determined following the study design described in Figure 1. The relative numbers of cell-associated genome equivalents of *C. burnetii* (GE) of NMI (**A**) and of NMII (**B**) were determined by *icd* qPCR. Significant differences between udder cells and other epithelial cell lines were determined by Mann–Whitney U test (a: *p* ≤ 0.05; b: *p* ≤ 0.01); L—Bel-26 (lung), Pm—BCEC (maternal placenta), Pf—F3 (fetal placenta), G—FKD-R 971 (jejunum), U—PS (udder).

Bovine epithelial cells from different tissues varied in their susceptibility to *C. burnetii* invasion and support of replication (Figure 2). In the experimental setting in which the inocula were left with the cells for 7 days (strategy A), higher numbers of genome equivalents (GE) of *C. burnetii* NMI and NMII were found cell-associated relative to the number in the regained inoculum (upper left graphs in Figures 2A and B; “invasion A”) only in the udder (U) epithelial cell line. The intestinal epithelial cell line (G) allowed moderate invasion of both NM strains. Regardless of the strain, very few bacteria were detected inside lung (L) and placental cells with fetal placental cells (Pf) containing more bacteria than maternal placental cells (Pm) which exhibited the lowest permissiveness for *C.* *burnetii* propagation throughout. Invasion rates in epithelial cell lines were not significantly different for NMI and NMII.

NMI and NMII, both showed a very low invasiveness within the first 24 h after inoculation (lower left graphs in Figures 2A and B; “invasion B”) under the experimental conditions applied. The udder epithelial cell line was only slightly more susceptible to incorporate *C. burnetii* than the lung and placental cell lines but differences did not reach statistical significance. Interestingly, although numbers markedly varied between biological replicates, intestinal epithelial cells exhibited the highest susceptibility for *C. burnetii* invasion within the first 24 h after inoculation.

Increases in *C. burnetii*-specific GE numbers during 7 days of culture following strategy A (upper right graphs in Figures 2A and B), reflect efficacy of *C. burnetii* replication but may also be influenced by invasion events having occurred later than 24 h. In order to dissect these two phenomena, excess, i.e., not yet cell-bound *C. burnetii* particles were removed 24 h after inoculation (“strategy B”; lower right graphs in Figures 2A and B). GE numbers were then quantified at day 7 and calculated relative to the numbers found cell-associated after 24 h of culture. It became apparent that udder epithelial cells were most effective in supporting replication of NMI and NMII (“replication B”): while numbers of NMI increased by approx. 16 000-fold from day 1 to day 7, numbers of NMII increased by approx. 330-fold (*p* = 0.032). Replication efficiency of NMI and NMII in intestinal epithelial cells was not significantly different from lung and maternal placental cells but fetal placental cells exhibited an intermediate support of *C. burnetii* replication.

### Large Coxiella containing vacuoles (CCV) were formed in gut and udder epithelial cells without affecting viability of the cell cultures

**Figure 3:**
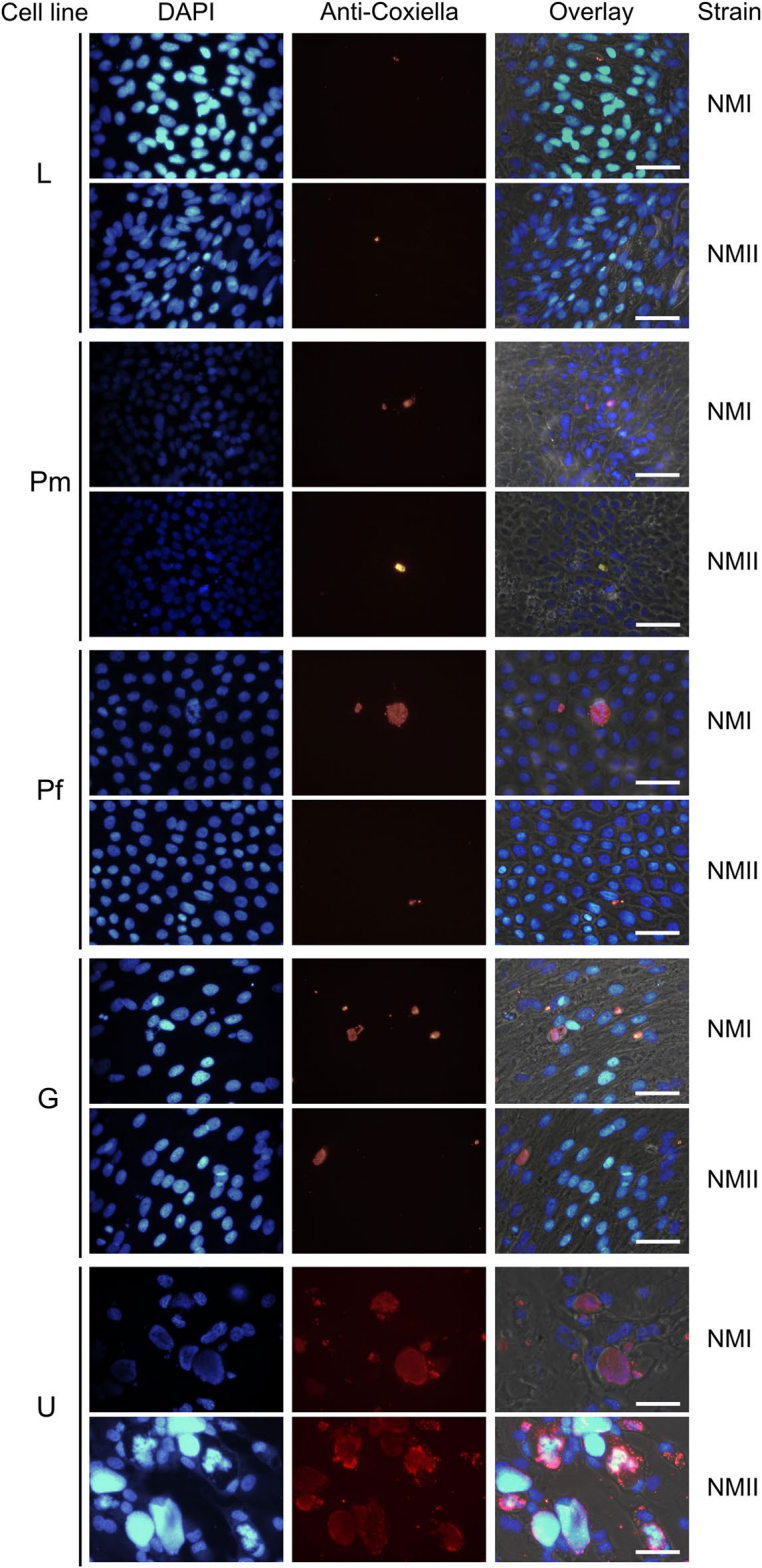
**Infection of bovine epithelial cells with** ***C. burnetii***. Fluorescence microscopy images showing bovine epithelial cells infected with *C. burnetii*-strains NMI and NMII. Nuclei were stained with DAPI (blue), bacteria were detected with an Anti-Coxiella-Alexa Fluor^®^ 594 labeled antibody combination (red). Microscopic pictures were taken 7 days after inoculation. White scale bars represent 50 µm length. Pictures are representative of three independent experiments.

**Figure 4:**
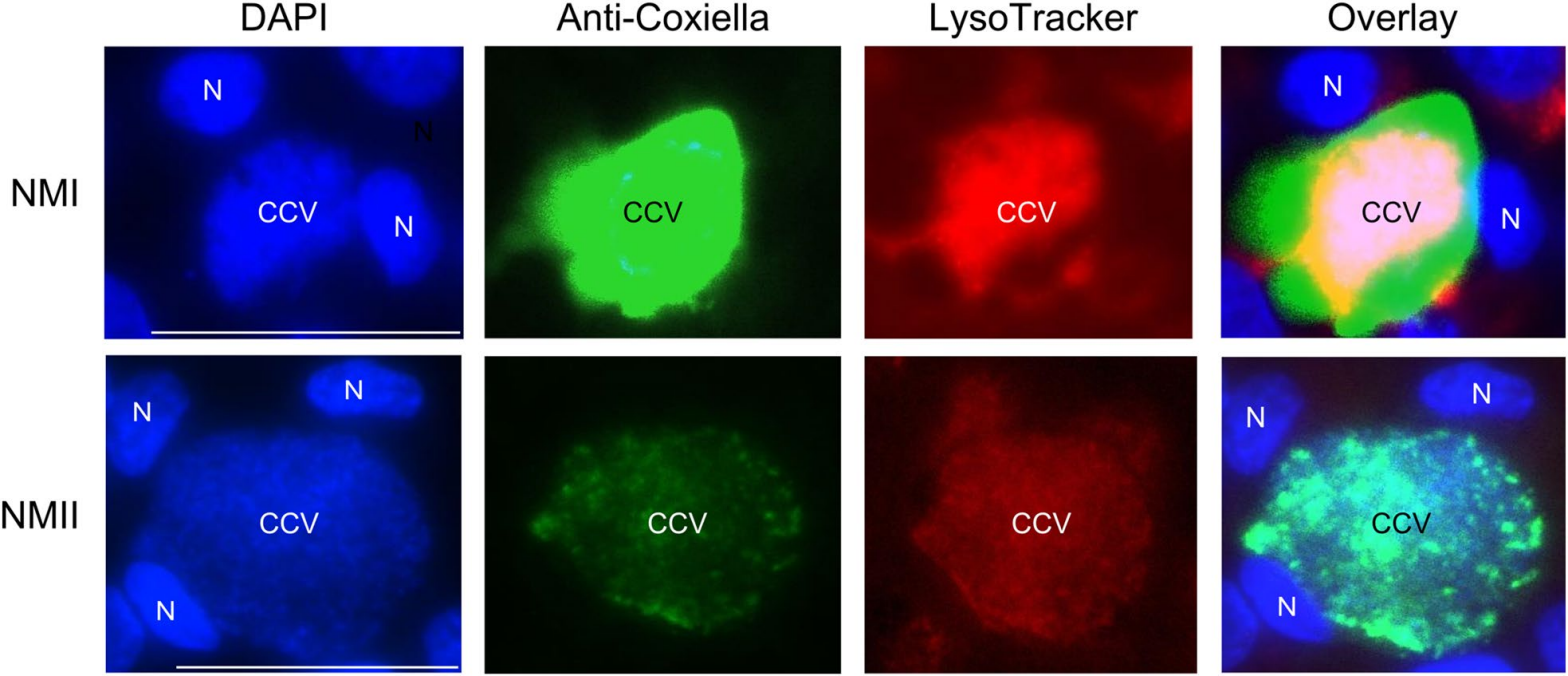
**Accumulation of LysoTracker red into** ***C. burnetii*** **containing vacuoles.** Bovine epithelial cells were infected with NMI and NMII for 7 days at 37 °C (strategy B_3_). Afterwards, cells were incubated for 2 h with LysoTracker red and additionally labeled with Anti-Coxiella antibody and DAPI and viewed by fluorescence microscopy. CCV identified by detection of *C. burnetii* (green) in vacuoles presents as acidified compartment (red) of the cell. N marks the nucleus labeled with DAPI (blue). White scale bars represent 50 µm length.

Immunofluorescence microscopy was applied to investigate the number and distribution of Coxiella organisms within epithelial cells 7 days after inoculation of monolayers (Figure 3). In corroboration of *icd* qPCR results, single cells, uniformly distributed within the monolayers of lung and placental epithelial cells harbored *C. burnetii* and bacterial accumulations were very small. Inoculation of cultures with NMI and NMII yielded comparable results. Monolayers of gut cells showed more infected cells but Coxiella clusters within cells also were small. The highest amount of bacterial accumulations was observed in udder cells, they were more closely spaced and filled up the whole cell. As reported for other *C. burnetii*-susceptible cell types, CCVs in udder epithelial cells induced by either of the *C. burnetii* strains presented as acidic compartments (Figure 4). In addition, small acidified vesicles were observed inside the infected cells next to the CCV (data not shown). A strong fluorescent signal of the LysoTracker Red dye inside the formed vacuoles indicated that phagosomal–lysosomal fusion had occurred.

**Figure 5:**
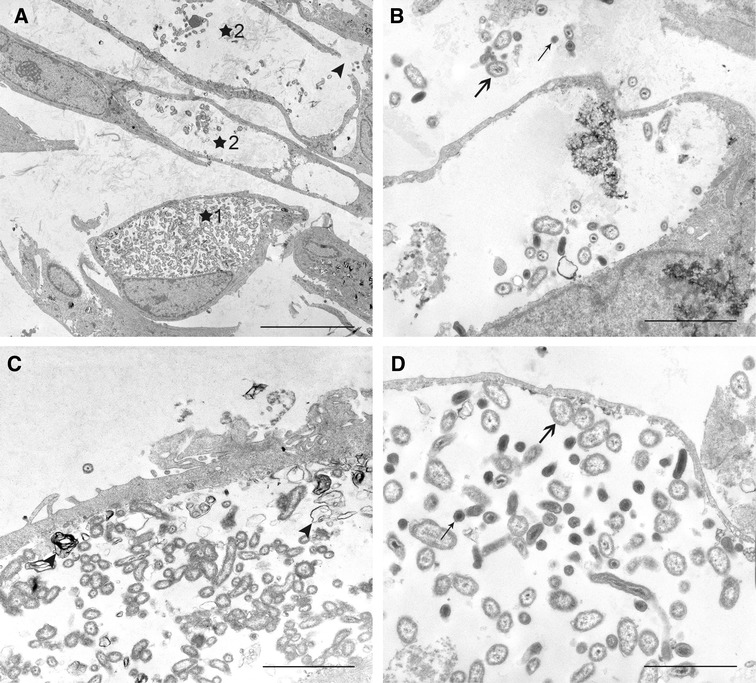
***C. burnetii*** **NMI and NMII in udder epithelial cells. A** CCV in udder cells filled with numerous (*1) or few (*2) *C.* *burnetii.* Note ruptured CCV (arrowhead). 7 days pi NMII. Bar = 8.0 µm. **B** Extracellular LCV (thick arrow) and SCV (thin arrow) adjacent to a large CCV. 7 days pi. NMI. Bar = 2.3 µm. **C** CCV containing predominantly LCV and small amounts of cellular debris (arrowheads). 7 days pi. NMII. Bar = 2.3 µm. **D** CCV filled with both LCV (thick arrow, exampl.) and SCV (thin arrow, exampl.). 7 days pi. NMII. Bar = 1.7 µm.

**Figure 6:**
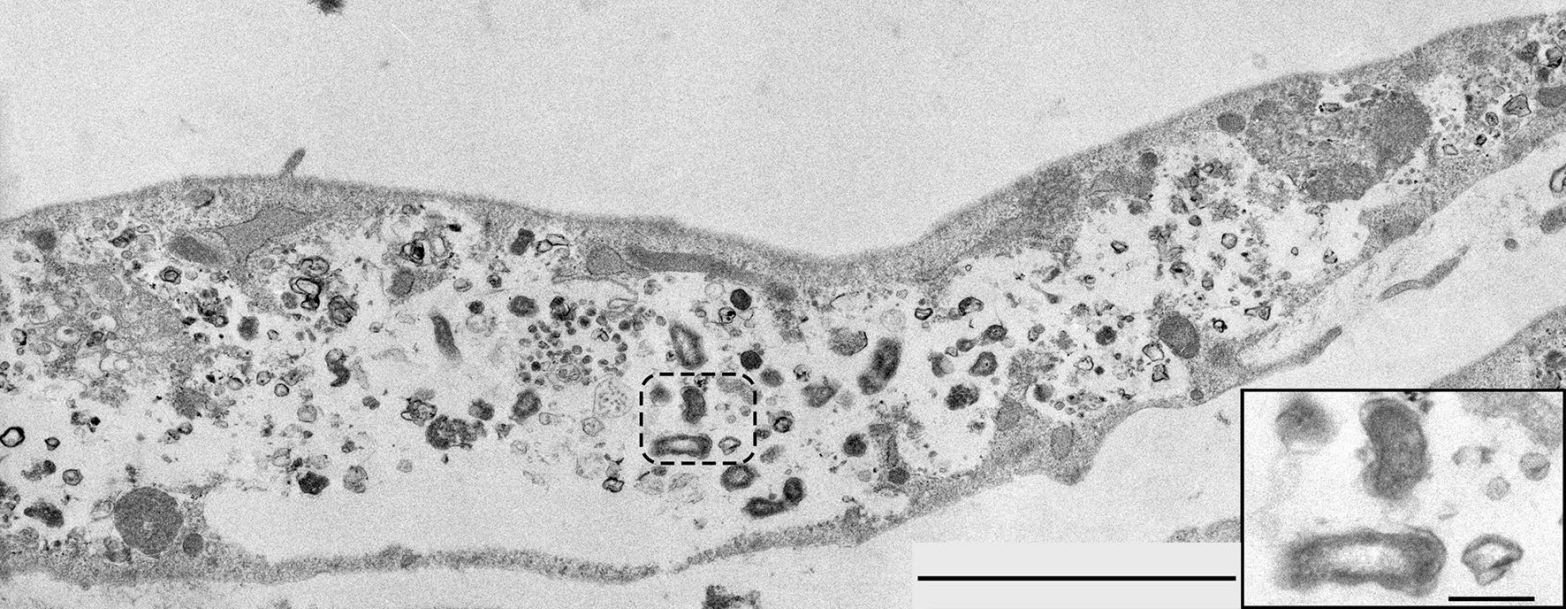
***C.*** ***burnetii*** **NMI in intestinal epithelial cells.** A large phagolysosome replaces most of the cytoplasm. It contains large amounts of cellular debris and few *C. burnetii* (inside hatched line, example). The area indicated by the hatched line is shown as inset. It contains LCV with the characteristic gram-negative cell wall. 7 days pi. NM1. Bar = 3.0 µm, bar inset = 300 nm.

By transmission electron microscopy numerous CCV were detected in udder and very few in intestinal cells at day 7 pi (Figures 5 and 6). Findings were comparable after infection with strains NMI and NMII. CCV were filled at a variable degree with large cell variants (LCV) representing the metabolically active form and small cell variants (SCV) representing the dormant form of *C. burnetii*. LCV had diameters of up to 400 nm and finely granular cytoplasma; SCV were about 150 nm in diameter, more electron dense and often had a highly electron dense central core. LCV and SCV were surrounded by a Gram-negative cell envelope formed by an inner membrane, a delicate cell wall, a periplasmic space and an outer membrane. The presence of both SCV as well as LCV indicates that *C. burnetii* undergoes a complete life cycle in udder and intestinal cells. CCV in udder cells were large and displaced most of the cytoplasm (Figure 5). Very large CCV which were often ruptured and contained few *C. burnetii* predominated. Since the cellular cytoplasm surrounding these CCV was very thin, their rupture may be artificial due to mechanical forces during processing. LCV and SCV in the extracellular space originated from the described CCV. Some CCV were densely packed with *C. burnetii*. Most of them were mainly filled with LCV, but in a few CCV both LCV and SCV were present. Small amounts of cellular debris were regularly admixed with *C. burnetii.* Intestinal epithelial cells were characterized by numerous, sometimes large phagolysosomes. Small numbers of LCV and SCV were present in very few of these phagolysosomes only (Figure 6). They were obscured by the large amounts of cellular debris. No extracellular *C. burnetii* were detected.

Epithelial cells infected with NMI or NMII retained their typical cell morphology after 7 days of infection and displayed no signs of cell death processes, e.g., disrupted cell membrane or fragmented nuclei. These observations could be confirmed by cell vitality assays. Independent of the *C. burnetii* strain, infection neither affected metabolic activity nor cytoplasmic membrane integrity of any of the epithelial cell lines after 1 and 7 days of inoculation compared to uninfected control cells (data not shown).

### CR3 and α_v_β_3_ surface expression does not correlate with epithelial susceptibility to *C. burnetii* invasion

**Figure 7:**
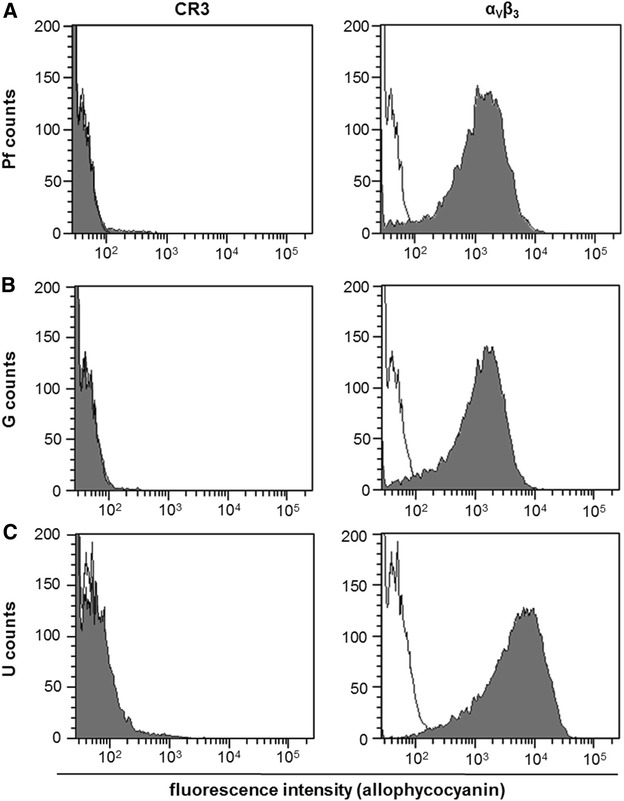
**Determination of receptor distribution on bovine epithelial cells.** Uninfected epithelial cell (Pf [panel **A**], G [**B**], U [**C**]) were analyzed by flow cytometry for expression of CR3 and α_V_β_3_ on their surface. Grey shaded curves depict detection of the respective antigens and black lines represent secondary antibody control (representative results of two technical replicates in two independent experiments).

Uptake of NMI is mediated by leukocyte response integrin (LRI α_v_β_3_) whereas the avirulent *C. burnetii* enters host cells through the combination of α_v_β_3_ and CR3 (complement receptor 3) [21]. To investigate whether differences in the susceptibility of bovine epithelial cells to *C. burnetii* invasion can be explained by varying cell surface expression pattern of these antigens, we applied flow cytometry analysis on fetal placental, intestinal and udder epithelial cells (Figure 7). All cells of the cell lines studied expressed α_v_β_3_. While fetal placental and intestinal epithelial cells expressed the integrin in comparable densities (as deduced from the distribution of fluorescence signals for the detection of the antigens), udder epithelial cells exhibited an enhanced α_v_β_3_ expression. Expression of CR3 was barely detectable on all cell lines. Expression pattern of these molecules therefore neither correlate with the enhanced susceptibility of intestinal epithelial cells for *C. burnetii* invasion nor with the fact that udder epithelial cells particularly supported *C. burnetii* replication rather than invasion.

### *C. burnetii* infection failed to induce a consistent inflammatory response in bovine epithelial cells

**Figure 8:**
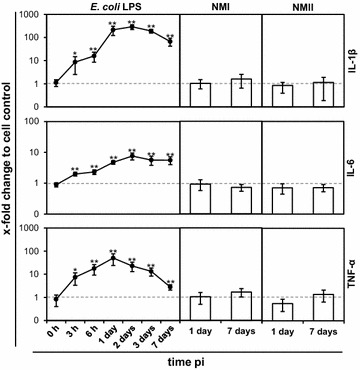
**Cytokine expression of udder epithelial cells after stimulation with** ***E. coli*** **LPS or infection with** ***C.*** ***burnetii***. Amounts of mRNA specific for IL-1β, IL-6 and TNF-α after *E. coli* LPS stimulation (5 µg/mL) or infection with NMI and NMII were measured by qPCR at the time points after inoculation of cultures as indicated. The data were normalized based on the housekeeping gene GAPDH and the unstimulated or uninfected cell control. A randomisation test with a pair-wise reallocation was used to compare ΔCT (cycle threshold)-values from four independent experiments (*p ≤ 0.05; **p ≤ 0.01).

**Figure 9:**
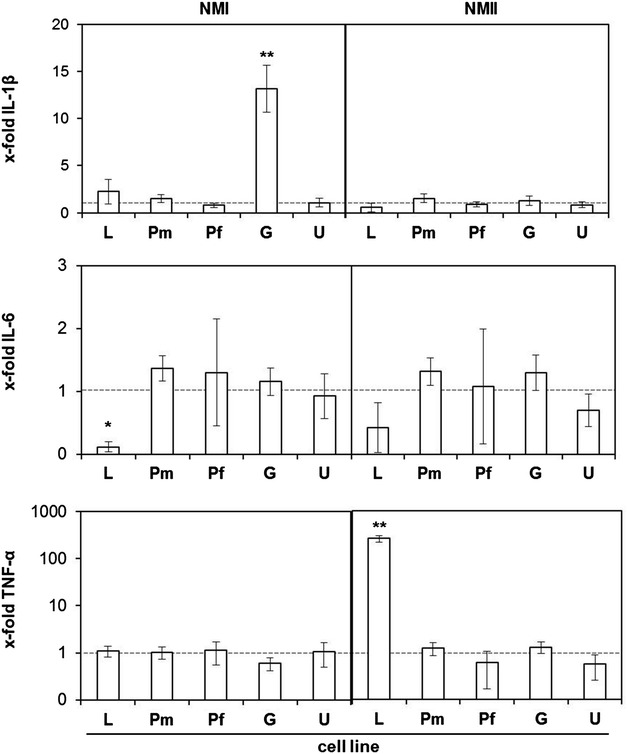
**Cytokine expression of epithelial cells after infection with** ***C.*** ***burnetii***. Amounts of mRNA specific for IL-1β, IL-6 and TNF-α 24 h after infection with NMI and NMII were measured by qPCR. The data were normalized based on the housekeeping gene GAPDH and the uninfected cell control. A randomisation test with a pair-wise reallocation was used to compare ΔCT (cycle threshold)-values from four independent experiments (*p ≤ 0.05; **p ≤ 0.01).

We previously showed that *C. burnetii* induces a short-lasting but pronounced pro-inflammatory cytokine response in bovine macrophages in the early phase of infection [13]. Stimulation of udder epithelial cells with *E. coli* LPS resulted in a significant increase in mRNAs specific for IL-1β, IL-6 and TNF-α, confirming the general ability of the cell line to respond to microbe-associated molecular patterns (Figure 8). Responses peaked at day 1 or day 2 and either declined upon prolonged stimulation (IL-1β, TNF-α) or remained elevated (IL-6). By stark contrast and despite the development of prominent CCVs in the udder epithelial cell line (see above), infection with neither NMI nor NMII induced an up-regulation of the transcription of the respective cytokines after 1 day and after 7 days of infection. Lung, placental and gut epithelial cells responded to stimulation with *E. coli* LPS with gradual increases of cytokine mRNA expression over a time period of 7 days (data not shown) but also poorly responded to infection with NMI and NMII at 1 day pi (Figure 9). Only gut epithelial cells specifically reacted to NMI infection with a significant upregulation of IL-1β, lung epithelial cells with a specific downregulation of IL-6 whereas infection with NMII resulted in a distinct up-regulation of TNF-α in lung epithelial cells.

## Discussion

The bovine epithelial cell lines utilized in this study were selected as surrogates of certain steps in the infection path *C. burnetii* follows inside a mammalian host. The lung represents the entry site for the pathogen to establish the infection with alveolar macrophages considered to play a key role [22]. Placenta, gut and udder are known replication sites immediately prior to transmission events [2]. Here we demonstrated that *C. burnetii* invaded and replicated in bovine epithelial cells from the different organs without destroying the cell integrity or inducing a substantial immune response. Although the *C. burnetii* strain NMI is considered more “virulent” than NMII as deduced from cell culture and rodent experiments, the invasiveness and replication efficacy in the bovine cell lines was similar. Epithelial cells from different organs differed in the individual kinetics of two steps in the cellular infection process. Udder epithelial cells were most effective in propagating *C. burnetii* mainly because of particularly supporting replication after bacterial invasion. Intestinal epithelial cells, by contrast, particularly supported bacterial invasion. The comparably higher replication efficacy detected for the latter cells when applying experimental strategy A as compared to strategy B points to ongoing invasion events even after 24 h of incubation which had paralleled bacterial replication. The interaction pattern with *C. burnetii* of these two cell types represented the extremes displayed by the cell lines under consideration with the suitability of lung, maternal and fetal epithelial cells to act as *C. burnetii* host cells ranging in-between.

Inoculation of bovine mammary gland epithelial cell cultures yielded the highest amounts of *C. burnetii* compared to cultures of epithelial cells from lung, gut and placenta after an incubation time of up to 7 days. Previous histological investigations of tissues from infected cattle primarily detected the pathogen in mammary gland epithelial cells (reviewed in [23]). Tropism for udder tissues in bovines seems to provide the basis for the high numbers of *C. burnetii* shed by milk in this species [9, 24, 25]. It is considered that consumption of dairy products poses a low risk for Q fever infections in humans [26] even though Benson et al. [27] had found that most bulk tank milk samples contained viable Coxiella organisms and human consumption of *C. burnetii*-containing milk leads to a rise in specific serum antibody titers in absence of clinical disease. A high bacterial load in raw milk was also described by Enright et al. [28], who observed up to 10 000 infective doses of *C. burnetii* per mL of milk from infected dairy cows. Similar bacterial load was observed by Schaal [29] after quantitative analysis of Coxiella containing milk. High numbers of *C. burnetii* in milk at least contribute to spreading of the agent within cattle herds. Newborn calves fed *C. burnetii*-containing milk excrete the bacteria in their feces and urine into the environment [3]. Therefore a dairy herd showing no symptoms of Coxiellosis could still be a *C. burnetii* reservoir for transmission via the tick-independent infection cycle [24].

Typical *C. burnetii* containing structures (CCV) were observed in udder and intestinal epithelial cells by transmission electron microscopy. Mature CCV were seen at 7 days pi in udder cells. Findings are comparable to findings in Vero cells [30, 31]. This indicates that both NMI and NMII undergo a complete replication cycle in these udder epithelial cells. The formation of large CCV requires protein secretion by *C. burnetii* [32] and depends on the actin cytoskeleton of the infected cells [33].

Small ruminants, like goats and sheep, shed *C. burnetii* more frequently via birth products [34] which contain huge numbers of bacteria and are the main source of environmental contamination and subsequent aerogenic transmission to humans. In ovine placentas, van Moll et al. [34] found Coxiella organisms in huge amounts in trophoblast cells which were embedded in acutely inflamed tissue. By contrast, bovine placentas generally contain few or moderate numbers of cells staining positive with Coxiella-specific antibodies and with rarely detectable Coxiella-like organisms. Our results of infection experiments with bovine fetal and maternal placental epithelial cell lines are in line with these ex vivo observations and imply that the many tissue-specific properties the placental cell lines have retained also comprise determinants of permissiveness for *C. burnetii* infection [35, 36].

Numbers of *C. burnetii* in the feces of infected small ruminants exceed numbers in bovine feces pointing to another possible shedding route at least in sheep and goats [9]. Feces of aborting ewes contain up to 10^7^ GE of Coxiella per gram [37]. Our in vitro study showed that epithelial cells other than mammary gland epithelium also were capable of internalizing *C. burnetii* but there was little further propagation of bacterial numbers within the cells. In organs implicated in *C. burnetii* shedding by cattle, these cells apparently represent rather unsuitable target cells which may be the in vitro correlate of the comparably low numbers of *C. burnetii* detected in the feces and birth fluids of bovines [9, 10].

At the entry site, lung epithelial cells are the first contact of Coxiella when entering the host organism after aerial transmission. It may be argued, that significant replication is not necessary at the entry site but the bacteria just cross the epithelial barrier to reach phagocytosing cells. Calverley et al. [38] concluded that recruited monocytes play an important role in the infection process because they control the distribution of bacteria from the lung. Additionally, resident alveolar immune cells were shown in a mouse model to possess a high susceptibility to Coxiella infection [38] and human and bovine alveolar macrophages can be infected with *C. burnetii* in vitro [13, 39]. There is cumulating evidence, therefore, that lung epithelial cells, being less susceptible to *C. burnetii* infection ([1], this study), do not act as replication sites for the bacteria and as such are poorly implicated in *C. burnetii* transmission between animals and in persistence in different hosts.

Bovine epithelial cells from different tissues varied in their susceptibility to *C. burnetii* invasion and support of replication with little correlation between the two properties as udder epithelial cells particularly supported *C. burnetii* replication whereas intestinal epithelial cells displayed an enhanced susceptibility for *C. burnetii* invasion. Invasion of *C. burnetii* into cells is reported to be influenced by the biochemical composition of the LPS [40]. In a variety of cells, *C. burnetii* strains with phase I and phase II LPS exhibit a different uptake kinetic in that a virulent phase I strain attached slower than the avirulent strain because of the mechanisms these organisms utilize to enter the host cells. Coxiella uses specific eukaryotic receptors such as integrins on macrophages and monocytes to adhere and invade [21]. *C. burnetii* phase I particles bind the leukocyte response integrin (α_v_β_3_), whereas the avirulent *C. burnetii* additionally deploy complement receptor 3 (CR3) [21]. Bovine udder epithelial cells exhibited an enhanced α_v_β_3_ expression whereas CR3 was essentially absent from the surface of all cell lines studied. Martinez et al. already described the first *C. burnetii* protein involved in host cell invasion [41]. OmpA is a surface protein of Coxiella that increased the internalization within non-phagocytic cells without necessity of Coxiella-specific receptors. Further investigations are required to assess the role of the adhesin OmpA and α_v_β_3_ for bacterial attachment, invasion and cellular activation in bovine epithelial cells.

Coxiella organisms can activate immune cells in a strain-dependent manner. Especially avirulent strains promote a higher pro-inflammatory cytokine production compared to virulent strains [39] which may be linked to LPS phase-related differences in attachment of the bacteria [42]. We observed a general failure to induce immune responses which particularly holds for udder epithelial cells independent of the phase-type of the NM variant and the day post-infection. Enterobacterial LPS is a very potent stimulant for immune reactions via pattern recognition receptors (PRRs) [43] and was included as positive control in our studies. LPS-stimulated udder epithelial cells showed an upregulation of pro-inflammatory cytokines. The failure of *C. burnetii* to initiate epithelial immune responses does not result from a process actively steered by a metabolically active pathogen, because infection studies with heat-inactivated NMI and NMII yielded similar results (data not shown). Invasive bacteria normally induce a rapid pro-inflammatory cytokine production as part of the defense mechanisms of the host. Different from bovine epithelial cells, attachment to or invasion of *C.* *burnetii* into macrophages stimulate a pro-inflammatory immune response to recruit additional immune cells [13, 39]. However, these responses are regulated in a complex manner. Rasmussen et al. [44] described a delay in cytokine expression in *Chlamydia* spp.-infected cells and that chlamydial invasion alone did not induce an immune response. In human colon epithelial cells, activation of cytokine response upon bacterial invasion is dependent on a special set of signals [45]. The attachment alone did not sufficiently stimulate the immune system for bacterial clearance. Activation of epithelial cells by *Candida albicans*, a common epidermal pathogen, is regulated via two phases of signal pathway activation [46]. On the one hand there is a morphological recognition of the fungus via PRRs and on the other hand a second trigger leads to production of cytokines and further immune reactions inside the host cells. The molecular basis for failure of bovine epithelial cells investigated in our study to initiate inflammatory responses remains to be determined. It can be assumed, though, that this property of *C. burnetii* is instrumental to create a replicative niche in the reservoir host for persistence of the pathogen, similar to what Ben Amara et al. [47] had suggested for human trophoblasts.

The in vitro cell system established and characterized in this study will be useful to further the understanding of the chain of events during the infection process of Coxiella organisms inside the bovine host in respect to epithelial cells as possible target cells. Bovine udder epithelial cells seem to have the highest permissiveness for *C. burnetii* and promote bacterial replication without losing the cell vitality. The udder cell line used was initially isolated from a mammary gland and became permanently cultivable by continuous passaging without deploying artificial transformation. The similarity to primary bovine mammary epithelial cells constitutes a big advantage for further investigations. Even though cell lines assessed in our study significantly varied in their permissiveness, the results strongly imply that *C. burnetii* can make use of epithelial cells beside immune cells as target cells for successful transmission between animals and into the environment as the cells survived the infection for substantial periods of time while helping the pathogen to evade the hosts immune response. For reasons of availability we chose bovine cells and evaluated the invasion and replication of *C. burnetii* by using two biological variants of a commonly used prototype strain. Cattle are not the main source of human infection but may shed *C. burnetii*. The high prevalence of *C. burnetii* genotype ST 20 recently identified in bovine milk in the U.S. [48] points to host species-specific adaptations within of the species of *C. burnetii*. After the proof-of-principle provided in this manuscript, studies are now in progress to assess quantitative differences in the interaction of different Coxiella genotypes with bovine epithelial cells.
